# An Infallible Cure for Tooth Ache

**Published:** 1848-07

**Authors:** 


### AN INFALLIBLE CURE FOR TOOTH ACHE.

Take of "Sands' Clove Anodyne," fifty cents worth, use it all; if the pain does not cease, try fifty cents worth of "Toothache Pills." They are easily obtained, and applied by at least *forty*, in the city of New York, whose whole dental knowledge is comprised in a single box, containing six of these *wonderful* "Pain Extractors." After the application of the "Tooth Ache Pill," if the pain increases, (a certainty in fifty cases in every hundred,) then apply a mustard plaster to the affected side of the face. Suffer with this four or five days. Should you then desire further evidence of human sufferings, we would refer you to a "brace" of worthy Dentists in New York, who say they are the "cheapest" Dentists in the United States, and that they fill teeth with 33 carat gold for THREE SHILLINGS each, and extract single teeth for ONE SHILLING. Where two are taken out at the same "pull," we suppose "One-and-Six" would be the price.

After all this suffering, in the language of the poet, we would say in a comforting manner:

"Thou haggard fiend, of hellish imps the worst,
To mercy deaf, by sorrowing man accurst;
Though cheerless days made desolate by thee,
And long long nights of sleepless agony,
Have marked thy fearful reign in days of yore,
Thy power is crushed—thy scorpion sting no more
Affrights the helpless, for the DENTAL ART,
Commands thy gloomy terrors to depart;
Then wipes from beautys cheek the tears that burn,
And bids her roses and her smiles return."

